# Nutritional and Phytochemical Potential of Two Local Food Plants in Burkina Faso: *Crateva adansonii* and *Capparis corymbosa*

**DOI:** 10.1002/fsn3.71272

**Published:** 2025-12-16

**Authors:** Edwige Noëlle Roamba, Frédéric Anderson Konkobo, Poussian Raymond Barry, Adama Pamba Séré, Abdoudramane Sanou, Roger Dakuyo, Kabakdé Kaboré, Kiessoun Konaté, Mamoudou Hama Dicko

**Affiliations:** ^1^ Laboratory Biochemistry, Biotechnology, Food Technology and Nutrition (LABIOTAN), Department of Biochemistry and Microbiology University Joseph KI‐ZERBO Ouagadougou Burkina Faso; ^2^ Applied Sciences and Technologies Training and Research Unit, Department of Biochemistry and Microbiology University of Dedougou Dedougou Burkina Faso; ^3^ Department Food Technology (DRO‐DTA)/IRSAT/CNRST Ouagadougou Burkina Faso

**Keywords:** antioxidant activity, *Capparis corymbosa*, *Crateva adansonii*, local food plants, nutritional characterization

## Abstract

This study focused on the biochemical and nutritional characterization of two edible plant species in Burkina Faso: *Capparis corymbosa* and *Crateva adansonii*. The objective was to evaluate their proximate composition, their content of primary and secondary metabolites, minerals, and amino acids, as well as their antioxidant potential, with a view to enhancing their nutritional value. The leaves of *Crateva adansonii* were found to be rich in protein (26.09 g/100 g dry matter [DM]), lipids (7.09 g/100 g DM), minerals, and energy value (297.45 kcal/100 g). The fruits of *Capparis corymbosa* stood out for their high carbohydrate content (33.04 g/100 g DM) and vitamin A content (0.87 mg/100 g DM). The amino acid profile revealed the presence of 18 to 19 amino acids, including 8 to 9 essential amino acids, highlighting their protein value. For bioactive compounds, the butanolic fractions of the leaves of both species had the highest concentrations of total polyphenols and flavonoids. The evaluation of antioxidant activity using the DPPH, ABTS, and FRAP methods showed a high capacity for free radical scavenging, particularly for macerated extracts (DPPH: 20.58 ± 0.72 μg/mL). *Crateva* leaves also had the highest anthocyanin content (2.1 g/100 g DM). In conclusion, *Crateva adansonii* and *Capparis corymbosa* appear to be promising plant sources, combining nutritional richness and high antioxidant potential. Their integration into the diet could contribute to improving nutritional security and the health of populations.

## Graphical abstract

*C. corymbosa* and *C. adansonii* contain many nutrients and secondary metabolites and antioxidants. These activities were evaluated by standard methods. These metabolites are a factor in the fight against various diseases.

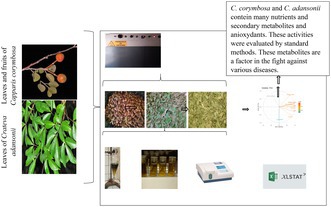

## 1 Introduction

Food plants play a crucial role in the nutrition and health of populations, particularly in regions where access to a diverse diet remains limited. One of the main nutritional challenges today is the inadequacy of diets, which results in deficiencies, excesses, or imbalances in energy and nutrients. These imbalances, observed in populations around the world, have serious repercussions on health, growth, and human development, contributing to the emergence of various forms of malnutrition and even premature death (Kahane et al. 2013; Romieu et al. 2017; Cardo et al. 2021). To meet these challenges, good nutrition is based on a diet that is both qualitative and quantitative, balanced and adapted to needs. Nutrition studies the functions of nutrients in the body, their interactions, and the nutritional requirements of individuals and population groups (Aranceta‐Bartrina et al. 2019; Lee et al. 2022). In this context, Burkina Faso has a rich and varied plant biodiversity, home to many local food and medicinal resources that are often underutilized or neglected. However, some of these species have particularly interesting nutritional and pharmacological potential (Masters 2021; Deme et al. 2021). Plants, as natural sources of phytonutrients, represent a promising avenue for enriching local diets while improving population health (Benítez et al. 2023). In certain regions of Burkina Faso, some species of plants from the *Capparidaceae* family deserve special attention due to their traditional use, ecological adaptability, and nutritional potential. Among these, *Capparis corymbosa* and *Crateva adansonii* stand out for their recurring culinary and medicinal uses, in addition to their resilience to harsh climatic conditions (Edwige et al. 2020). Indeed, *Capparis corymbosa* , commonly found in termite mound vegetation, plays an important role in traditional food systems in Burkina Faso. This shrub species is well known to rural populations, who use its various parts for food and medicinal purposes. Its leaves and fruits are commonly eaten boiled or incorporated into local sauces, often accompanied by cereals such as millet or sorghum. In the Central, Central Plateau, and Northern regions, where access to cultivated vegetables may be limited during the dry season, *C. corymbosa* provides a valuable alternative source of micronutrients and bioactive compounds. *Crateva adansonii* is also a plant species belonging to the *Capparidaceae* family, well known for its medicinal properties and traditionally used to treat various health problems (Rathinavel et al. 2021). This species is found in the Sahelian and Sudanian zones, where it grows mainly on light soils along riverbanks. From a culinary perspective, the young leaves of *Crateva adansonii* are often prepared in thick sauces or combined with other leafy vegetables in popular local dishes, particularly in the southwestern regions of Burkina Faso. Appreciated for their texture and slightly bitter taste, they are considered nutritious and beneficial to health. The objective of this study is to evaluate the biochemical and nutritional composition, as well as the antioxidant activity of the edible parts of *Capparis corymbosa* (leaves and fruits) and *Crateva adansonii* (leaves). By characterizing these two species, our approach aims to contribute to the promotion of local food biodiversity while supporting a sustainable nutrition approach adapted to the ecological contexts of Burkina Faso and sub‐Saharan Africa. This study also aims to provide a solid scientific basis for encouraging the integration of these two plants into local food systems and, potentially, into other regions facing similar challenges in terms of nutritional security and food resilience.

## 2 Materials and Methods

### 2.1 Sample Collection

**FIGURE 1:**
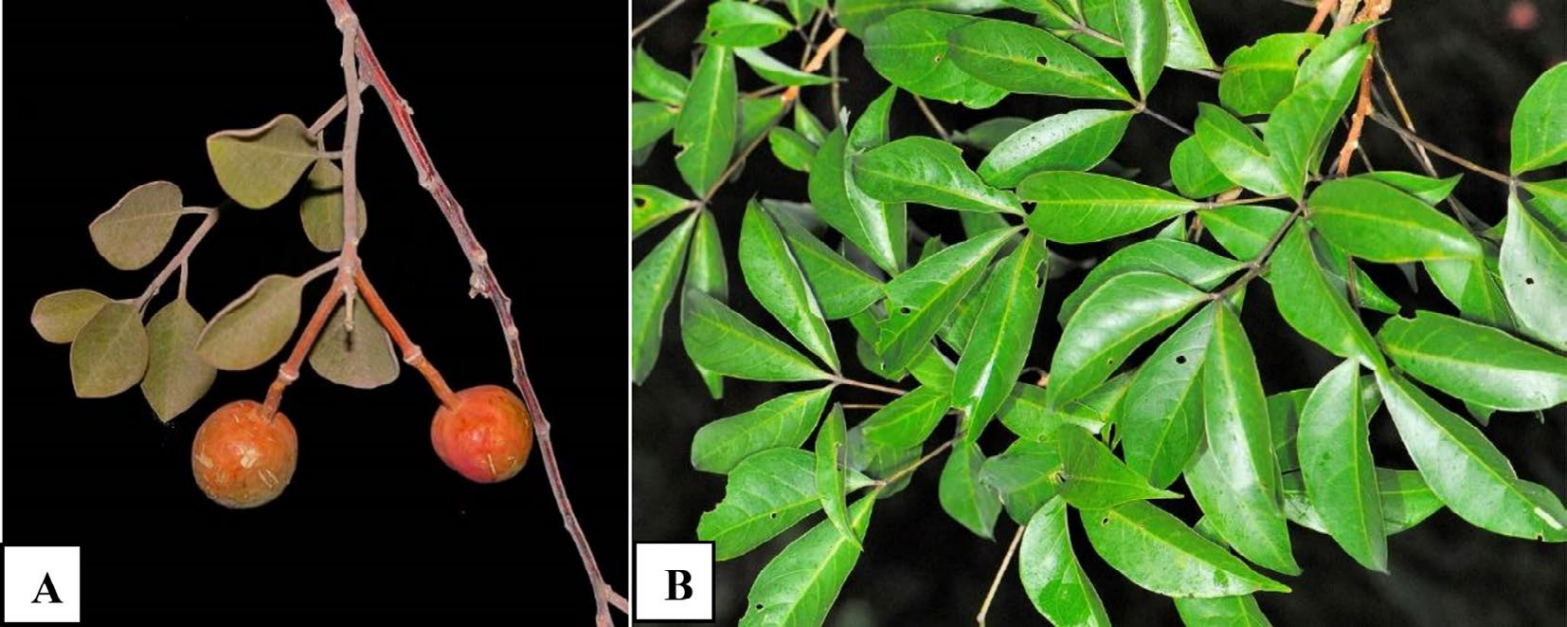
Leaves and fruits of *Capparis corymbosa* (A). Leaves of *Crateva adansonii* (B).

The leaves and fruits of *Capparis corymbosa* and *Crateva adansonii* (Figure 1) were collected in August 2023 in the rural commune of Fara, located in the Boucle du Mouhoun region of Burkina Faso. After collection, the samples were transported to the laboratory where they were sorted, washed, and then dried in the shade in a well‐ventilated area to avoid degradation of thermolabile and photosensitive bioactive compounds, thereby preserving phenolics, flavonoids, and other secondary metabolites that could be altered during high‐temperature drying. Once dry, the different samples were ground separately to obtain a fine powder, which was stored in airtight jars at room temperature, protected from light and moisture, pending analysis.

### 2.2 Determination of the Proximal Composition

#### 2.2.1 Determination of Water and Dry Matter Content

The water content was determined using the method described by (Konkobo et al. 2025). A 5 g sample was placed in a pre‐weighed crucible and then placed in an oven (Binder, Tuttlingen, Germany) at 105°C for 4 h. After heating, the crucible was transferred to a desiccator (Kartell, Milan, Italy) to cool for 30 min. This operation was repeated until a constant weight was obtained. The water content was then expressed in grams per 100 g of dry matter (g/100 g DM).

The DM content was deduced from the water content using the following formula: % DM = 100% − % *W*, where DM represents the DM content and *W* represents the water content.

#### 2.2.2 Determination of Total Ash

The ash content was determined using the method described by (Konkobo et al. 2025). To do this, a 5 g sample was placed in pre‐weighed crucibles and then incinerated in a muffle furnace (Nabertherm L5/11, Lilienthal, Germany) at 550°C for 5 h until white ash was obtained. After cooling in a desiccator (Kartell, Milan, Italy), the ash content was calculated by difference in mass.

#### 2.2.3 Determination of Titratable Acidity

Titratable acidity was determined using the colorimetric method described by (Bazie et al. 2022). To do this, 5 g of each sample was mixed with 50 mL of distilled water. After stirring for 5 min using a magnetic stirrer (Hanna Instruments HI300N, Woonsocket, RI, USA), the mixture was centrifuged (Sigma 3‐16P, Osterode am Harz, Germany) at 4000 g for 20 min. 10 mL of the supernatant was then taken, and a few drops of phenolphthalein (Sigma‐Aldrich, St. Louis, MO, USA) were added. Finally, the mixture was titrated with a sodium hydroxide solution (NaOH 0.1 N, Sigma‐Aldrich, St. Louis, MO, USA; Cat. No. S5881) until a pink color appeared, indicating the equivalence point.

### 2.3 Determination of Macromolecule Composition

#### 2.3.1 Determination of Carbohydrates

Carbohydrates were determined using the phenol‐sulfuric acid colorimetric method described by (Dakuyo, Konaté, Bazié, et al. 2022). To do this, a volume of 300 μL of sample was mixed with 150 μL of 5% phenol (Sigma‐Aldrich, St. Louis, MO, USA; Cat. No. P4682), then 750 μL of concentrated sulfuric acid (Sigma‐Aldrich, St. Louis, MO, USA; Cat. No. 339741) was added. After stirring, the mixture was incubated in a water bath (Grant Instruments, Cambridge, UK) at room temperature for 20 min. A blank was prepared with 80% ethanol (Carlo Erba Reagents, Val‐de‐Reuil, France) instead of the sample. The absorbance was measured at 490 nm using a spectrophotometer (Epoch, BioTek Instruments Inc., Winooski, VT, USA). The total carbohydrate concentration was determined using a glucose calibration curve, and the results were expressed in g/100 g of DM.

#### 2.3.2 Determination of Protein Content

Total proteins were quantified using the Bradford method described by (Konkobo et al. 2025). The extraction solution was prepared by dissolving 7.5 g of sodium dodecyl sulfate (SDS, Rectapur, VWR, Fontenay‐sous‐Bois, France; Cat. No. 43620.260) in 500 mL of distilled water, then adding 225 μL of β‐mercaptoethanol (Labosi, Paris, France). Next, 500 mg of powder from each sample was homogenized in 12.5 mL of this solution, followed by centrifugation (Sigma 3‐16P, Osterode am Harz, Germany) at 4500 rpm for 15 min. The supernatant obtained was used for the assay. To do this, a volume of 50 μL of supernatant was mixed with 50 μL of distilled water and 250 μL of Bradford reagent (Sigma Life Science, St. Louis, MO, USA; Cat. No. B6916). After incubation for 5 min at room temperature, the absorbance was measured at 595 nm using a spectrophotometer (Epoch, BioTek Instruments Inc., Winooski, VT, USA). The protein concentration was determined from a calibration curve established with bovine serum albumin (BSA, 0–250 μg/mL, Sigma‐Aldrich, St. Louis, MO, USA; Cat. No. A7906). The results were expressed in grams of BSA equivalent per 100 g of DM (g E BSA/100 g DM).

#### 2.3.3 Determination of Lipid Content

The lipid content was determined by hexane (Carlo Erba Reagents, Val‐de‐Reuil, France; Cat. No. 412371) extraction using the AOAC (1995) gravimetric method, with a Soxhlet apparatus (Gerhardt Soxtherm, Königswinter, Germany). To do this, a 5 g sample was acidified, filtered, and then the filtrate was dried and placed in an extraction cartridge lined with cotton wool. This was placed in the Soxhlet apparatus, and extraction was carried out with 200 mL of boiling hexane. The extracted lipids were recovered using a rotary evaporator (Heidolph Laborota 4000, Schwabach, Germany) and then weighed after evaporation of the solvent. The lipid content was calculated by mass difference and expressed in g/100 g of DM according to the following formula:

$$\text{Content}\;\left(g/100\;g\;\mathrm{DM}\right)=\left(P_{f}-P_{o}\right)/\mathrm{PE}\times 10$$

#### 2.3.4 Determination of Energy Value

The potential energy value was estimated using Coleman's formula, described by using Atwater's coefficients (Kaboré et al. 2022). It was calculated from the macronutrient content using the following equation:

$$\text{Energy}\;\left(\text{kcal}/100\;g\right)=\left(P\times 4\right)+\left(G\times 4\right)+\left(L\times 9\right)$$

where *P*, *G*, and *L* represent the protein, carbohydrate, and lipid contents (in g/100 g DM), respectively.

#### 2.3.5 Determination of β‐Carotene Content

The β‐carotene content was determined using the method described by (Bazie et al. 2022). A dry sample (100 mg) was extracted with 10 mL of an acetone/hexane mixture (4:6, v/v, Sigma‐Aldrich, St. Louis, MO, USA; Cat. Nos. 34850 and 412371) by vigorous shaking for 1 min, then filtered using Whatman No. 4 paper (Cytiva, Maidstone, UK). The absorbance of the filtrate was measured at 453, 505, and 663 nm using a spectrophotometer (Epoch, BioTek Instruments Inc., Winooski, VT, USA). The analyses were performed in triplicate. The β‐carotene concentration, expressed in mg/100 g of dry sample, was calculated using the following formula:

$$\begin{array}{cc} \beta -\text{carotene}\;\left(\mathrm{mg}/100\;\mathrm{mL}\right)\;=\; & 0.216\times A663-0.304\\ & \times A505+0.452\times A453 \end{array}$$

### 2.4 Amino Acid Determination

Amino acids were determined using the Waters PICO‐TAG method described by (Kaboré et al. 2022). This method was carried out in three stages: hydrolysis of each sample with 6 N HCl at 110°C for 24 h; derivatization of amino acids with phenylisothiocyanate (PITC, Sigma‐Aldrich, St. Louis, MO, USA; Cat. No. 139742‐100G, ≥ 98%, reagent grade); and analysis of phenylthiocarbamyl (PTC) derivatives by reverse‐phase high‐performance liquid chromatography (HPLC). For the analysis, 500 mg of powder from each defatted sample was hydrolyzed, then the mixture was filtered and derivatized. To the derived sample, 200 μL of PICO‐TAG dilution solution (0.38 μg/μL, Waters Corporation, Milford, MA, USA; Cat. No. WAT088119) was added, and the analysis was performed by HPLC (2.3 μg/μL) with detection at 254 nm. This method allowed the detection of up to 1 picomole of amino acid.

The percentage of each amino acid (% aa) was calculated using the following formula:

$$\%\mathrm{aa}=\left[\left(\text{Amino acid concentration}\right)/\left(\text{Sample concentration}\right)\right]\times 100$$

### 2.5 Mineral Analysis

The mineral content was determined by flame atomic absorption spectrometry (FAAS) according to the method described by. Mineral extraction was performed after calcination of 3 g of ground material from each sample in a muffle furnace (BINDER, Model ED 53, Tuttlingen, Germany) at 550°C for 24 h. The ash obtained was dissolved in 5 mL of concentrated hydrochloric acid (HCl, ≥ 37%, Sigma‐Aldrich, St. Louis, MO, USA, Cat. No. 30721) followed by the addition of three drops of hydrogen peroxide (H_2_O_2_, 30%, Sigma‐Aldrich, St. Louis, MO, USA, Cat. No. 216763). The mixture was transferred to a 100 mL volumetric flask (PYREX, Corning, NY, USA, Cat. No. 4980) and topped up with Milli‐Q water. After centrifugation, the supernatant was collected in sterile tubes (SARSTEDT, Nümbrecht, Germany, Cat. No. 72.692.250) for analysis. Mineral concentrations were measured using a flame atomic absorption spectrophotometer (Analytik Jena, contrAA 700, Jena, Germany) connected to a computer.

### 2.6 Determination of Phenolic Compounds and Antioxidant Activity

#### 2.6.1 Extraction

##### 2.6.1.1 Decoction

A mass of 10 g of dry powder was added to 200 mL of distilled water contained in a flask (PYREX, Corning, NY, USA, Cat. No. 4980). The mixture was heated to 80°C for 30 min. The decoction was filtered three times using Whatman paper (Cytiva, Maidstone, UK). The same process was repeated twice on the residue. The aqueous extract obtained was concentrated under vacuum using a rotary evaporator (Büchi Rotavapor R‐300, Flawil, Switzerland) at 80°C until a crude aqueous extract was obtained.

##### 2.6.1.2 Maceration

A test sample of 50 g of powdered *Crateva adansonii* leaves and *Capparis corymbosa* leaves and fruits was subjected to extraction under magnetic stirring (Hanna instrument, Model HI 190, Woonsocket, RI, USA) at room temperature in the laboratory for 48 h with 500 mL of a mixture of acetone (80%, Sigma‐Aldrich, Cat. No. 179124)—water (80:20) (I C S, 2008.). After maceration, the extract was filtered through Whatman paper. After filtration, the crude extracts obtained were subjected to evaporation in an oven (BINDER, Model ED 53, Tuttlingen, Germany) at 40°C. The dried hydroacetonitrile extract was homogenized in distilled water and filtered using Whatman paper. The filtrate obtained was then placed in a separating funnel for fractionation using different immiscible organic solvents with increasing polarities: hexane (≥ 99%, Sigma‐Aldrich, Cat. No. 11054‐3), dichloromethane (DCM) (≥ 99.8%, Sigma‐Aldrich, Cat. No. 270997), ethyl acetate (≥ 99.5%, Sigma‐Aldrich, Cat. No. 319902), butanol (≥ 99.5%, Sigma‐Aldrich, Cat. No. 34867).

The fractionation yield was calculated using the formula: ER = (*m*/*M*) × 100, where ER: extraction rate; *m*: mass of the extracted residue; *M*: mass of the plant powder.

##### 2.6.1.3 Fractionation of Extracts by Liquid–Liquid Separation

###### 2.6.1.3.1 Hexane Fraction

Fifty milliliter of the hydroacetonitrile macerate was extracted with 2 × 50 mL of n‐hexane (n‐H). The petrol ether extracts obtained were collected and then concentrated under reduced pressure, yielding the hexane fractions.

###### 2.6.1.3.2 Dichloromethane Fraction

The hexane‐exhausted aqueous phases were re‐extracted as indicated above, but with 2 × 50 mL of DCM as the solvent. The extracted solutions obtained were collected, concentrated, and dried to give the DCM fractions (FDCM).

###### 2.6.1.3.3 Ethyl Acetate Fraction

The aqueous phases exhausted after the two treatments were extracted again with 2 × 50 mL of ethyl acetate as solvent. The extracts obtained were combined, concentrated, and dried to give the ethyl acetate fraction.

###### 2.6.1.3.4 Butanol Fraction

The aqueous phases from the three previous treatments were extracted again with 2 × 50 mL of n‐butanol. The butanol extracts were collected, and the solvent was concentrated and dried in an oven to give the butanol fraction.

#### 2.6.2 Determination of Phenolic Compounds

Polyphenols were quantified using the Folin–Ciocalteu method by (Barry et al. 2022). This method is based on the reduction of the Folin–Ciocalteu reagent (Sigma‐Aldrich, St. Louis, MO, USA, Cat. No. 47641‐100mL) by phenolic compounds, forming a blue complex that can be measured by spectrophotometry. For the analysis, 60 μL of extract (obtained from 0.04 g of dry extract dissolved in 10 mL of DMSO) was diluted and used in place of gallic acid (≥ 98%, Sigma‐Aldrich, St. Louis, MO, USA, Cat. No. G7384), which was used as a standard. The contents were determined from the calibration curve and expressed in milligrams of gallic acid equivalent per 100 mg of sample (mg EAG/100 mg).

The total flavonoid content was determined using the aluminum trichloride (AlCl_3_, ReagentPlus, 99%, Sigma‐Aldrich, St. Louis, MO, USA, Cat. No. 237051) colorimetric method, according to the Dowd method, adapted by (Sultana et al. 2024). This method is based on the formation of a yellow complex between AlCl_3_ and the oxygenated groups of flavonoids contained in the samples. To do this, a mixture of 0.75 mL of extract from each sample and 5 mL of methanol without AlCl_3_ was used as a blank. The results were obtained from the calibration curve and expressed in milligrams of quercetin equivalent per 100 mg of extract (mg EQ/100 mg).

#### 2.6.3 Determination of Antioxidant Activity

The antioxidant activity of the extracts was evaluated using three complementary methods: DPPH, FRAP, and ABTS, using ascorbic acid as a standard. The results were expressed in mg ascorbic acid equivalent per gram of dry extract (mg AAE/g).

##### 2.6.3.1 DPPH Test

The free radical scavenging capacity was determined according to the method described by (Musa et al. 2016). To do this, a DPPH (2,2‐Diphenyl‐1‐picrylhydrazyl, Sigma‐Aldrich, St. Louis, MO, USA, Cat. No. D9132) solution (20 mg/L in 80% ethanol) was mixed with a sample solution (1 mg/mL). After 15 min of incubation in the dark, the absorbance was measured at 517 nm. The discoloration observed is proportional to the antioxidant activity.

##### 2.6.3.2 Reducing Power (FRAP)

The antioxidant reducing power was evaluated according to the method based on the reduction of potassium ferricyanide complex to ferrous iron (Fe^2+^) described by (Maliar et al. 2023). After incubation with (FeCl_3_, 0.1%, Merck, Darmstadt, Germany) the absorbance was measured at 700 nm. The intensity of the blue‐green color reflects the reducing capacity of the extracts.

##### 2.6.3.3 ABTS Method

The neutralization capacity of the radical cation ABTS^+^ was determined according to the method described by (Olszowy and Dawidowicz 2018). A freshly prepared ABTS^+^ solution (FLUKA Biochimika, Germany) was added to the extracts (1 mg/mL), and after 15 min of incubation in the dark, the absorbance was measured at 734 nm. The discoloration of the chromophore radical indicates the antioxidant efficacy of the sample.

### 2.7 Determination of Anti‐Nutritional Factors

#### 2.7.1 Determination of Phytate Content

Phytates were extracted by incubating 250 mg of sample with 10 mL of 2.4% HCl (≥ 37%, analytical grade, Sigma‐Aldrich, Cat. No. 320331) under agitation for 3 h at room temperature, then centrifuged at 6000 rpm (Eppendorf 5810 R, rotor F‐34‐6‐38) for 20 min (Dakuyo, Konaté, Sanou, et al. 2022). The supernatant was purified on an anion exchange resin. The assay was performed by measuring the absorbance at 500 nm after reaction with FeCl_3_/sulfosalicylic acid (Iron (III) chloride hexahydrate, ≥ 98%, Sigma‐Aldrich, Cat. No. 236489)/sulfosalicylic acid (≥ 99%, Sigma‐Aldrich, Cat. No. S1888). Phytic acid was used as a standard (≥ 98%, Sigma‐Aldrich, Cat. No. P8810).

#### 2.7.2 Determination of Tannins Content

Condensed tannins were determined using a colorimetric method based on the reaction between vanillin (≥ 99%, Sigma‐Aldrich, Cat. No. V1104) and the terminal units of tannins in an acidic medium (Li et al. 2023). To do this, a stock solution of the extract from each sample was prepared at 1 mg/mL in methanol (≥ 99.9%, HPLC grade, Sigma‐Aldrich, Cat. No. 34860). A volume of 400 μL of this solution was mixed with 3 mL of 4% (w/v) vanillin, then with 1.5 mL of hydrochloric acid (HCl, ≥ 37%, analytical grade, Sigma‐Aldrich, Cat. No. 320331). The mixture was incubated at 30°C for 20 min in the dark, and the intensity of the colored complex formed was measured by spectrophotometry at 500 nm (Epoch, BioTek Instruments Inc., Winooski, VT, USA).

The hydrolyzable tannin content was determined according to the protocol of (Carboni Martins et al. 2022), based on the formation of a colored complex between hydrolyzable phenolic compounds and ferric chloride. An aliquot of the extract from each sample (5 mg/mL) was mixed with 3.5 mL of a 0.01 M FeCl_3_ solution (Iron (III) chloride hexahydrate, ≥ 98%, Sigma‐Aldrich, Cat. No. 236489). After 15 min of reaction at room temperature, the absorbance was measured at 660 nm.

### 2.8 Determination of Anthocyanin Content

The anthocyanin content was determined using the differential pH method, as described by (Taghavi et al. 2022). This method is based on the variation in the color of anthocyanins depending on pH. A volume of 1 mL of lyophilized aqueous extract was mixed separately with 4 mL of two buffers, buffered at pH 1.0 and pH 4.5. After 15 min of incubation at room temperature, the absorbances were measured at 510 and 700 nm using a spectrophotometer (Epoch, BioTek Instruments Inc., Winooski, VT, USA). The total anthocyanin concentration was expressed in cyanidin‐3‐o‐sambubioside equivalents (EC/L), using the formula:

$$\text{Total anthocyanins}=\left(A\times \mathrm{MW}\times V\times \mathrm{DF}\right)/\left(\varepsilon \_\text{mole}\times L\right)$$

where *A*: absorbance = [(*A*_510_ − *A*_700_)] pH_1.0_ − [(*A*_510_ − *A*_700_)] pH_4.5_; MW: molecular weight; DF: dilution factor; *ε*: molar extinction coefficient of cyanidin‐3‐glucoside (26,900 M^−1^ cm^−1^) and *L* = path length (cm).

### 2.9 Statistical Analysis

Data were statistically analyzed using XLSTAT software. Analysis of variance (ANOVA) was performed, and mean comparisons were carried out using Tukey's test at the 5% significance level. Principal component analysis (PCA) was also conducted to explore relationships among variables. All experimental measurements were performed in at least triplicate, and results are expressed as mean ± standard deviation (SD).

## 3 Results

### 3.1 Physicochemical Composition

**FIGURE 2:**
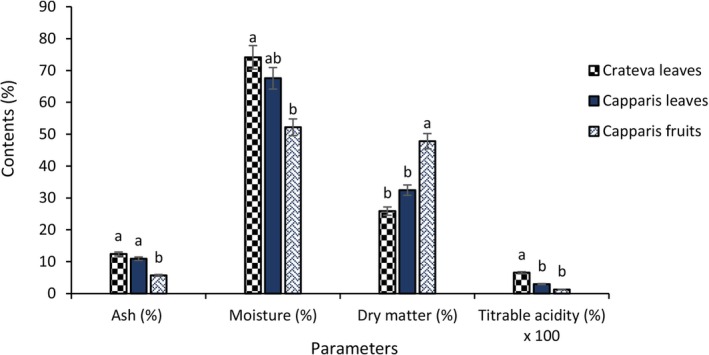
Physicochemical composition of *Crateva* leaves and *Capparis* fruits and leaves. The letters “a, b” indicate the different variations for each parameter. Sticks with the same letters do not have statistically different values. However, sticks with different letters have statistically different values.

Figure 2 illustrates the physicochemical composition of *Crateva* leaves, as well as *Capparis* leaves and fruits. It shows that fresh *Crateva* leaves have the highest water content (74.12%) and ash content (12.40%), but also the lowest DM content (25.88%). In contrast, *Capparis* fruits are characterized by a high DM content (47.81%) combined with a more moderate water content (52.19%). *Capparis* leaves have intermediate levels for all three parameters. Finally, in terms of titratable acidity, *Crateva* leaves are slightly more acidic (0.12%) than *Capparis* leaves and fruits (0.010% and 0.05%, respectively).

### 3.2 Nutritional Composition

**FIGURE 3:**
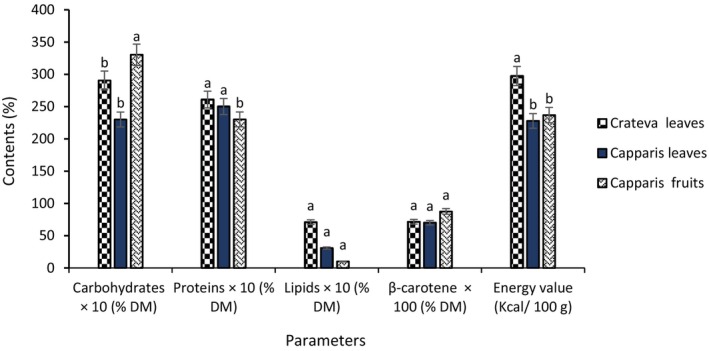
Nutritional composition of *Crateva* leaves and *Capparis* fruits and leaves. The letters “a, b” indicate the different variations for each parameter. Sticks with the same letters do not have statistically different values. However, sticks with different letters have statistically different values.

Figure 3 shows the main nutrient contents of *Crateva* leaves and *Capparis* leaves, and fruits. These data highlight the differences in nutritional profiles between the species studied. Carbohydrates are found mainly in *Capparis* fruit (33.04 g/100 g DM), followed by *Crateva* leaves (29.04 g/100 g DM) and *Capparis* leaves (230 g/100 g DM). Protein is higher in *Crateva* leaves (26.09 g/100 g DM), with similar levels in *Capparis* leaves (25.01 g/100 g DM) and fruits (23.01 g/100 g DM). In terms of lipids, *Crateva* leaves have a notable content (7.09 g/100 g DM), slightly higher than that of *Capparis* leaves (3.09 g/100 g DM) and fruits (1.01 g/100 g DM). Vitamin A is also more concentrated in *Capparis* fruit (0.87), followed by *Crateva* leaves (0.71) and *Capparis* leaves (0.70). Finally, the energy value is highest in *Crateva* leaves (297.45 kcal/100 g), compared to 236.96 kcal/100 g for fruits and 227.82 kcal/100 g for *Capparis* leaves.

**FIGURE 4:**
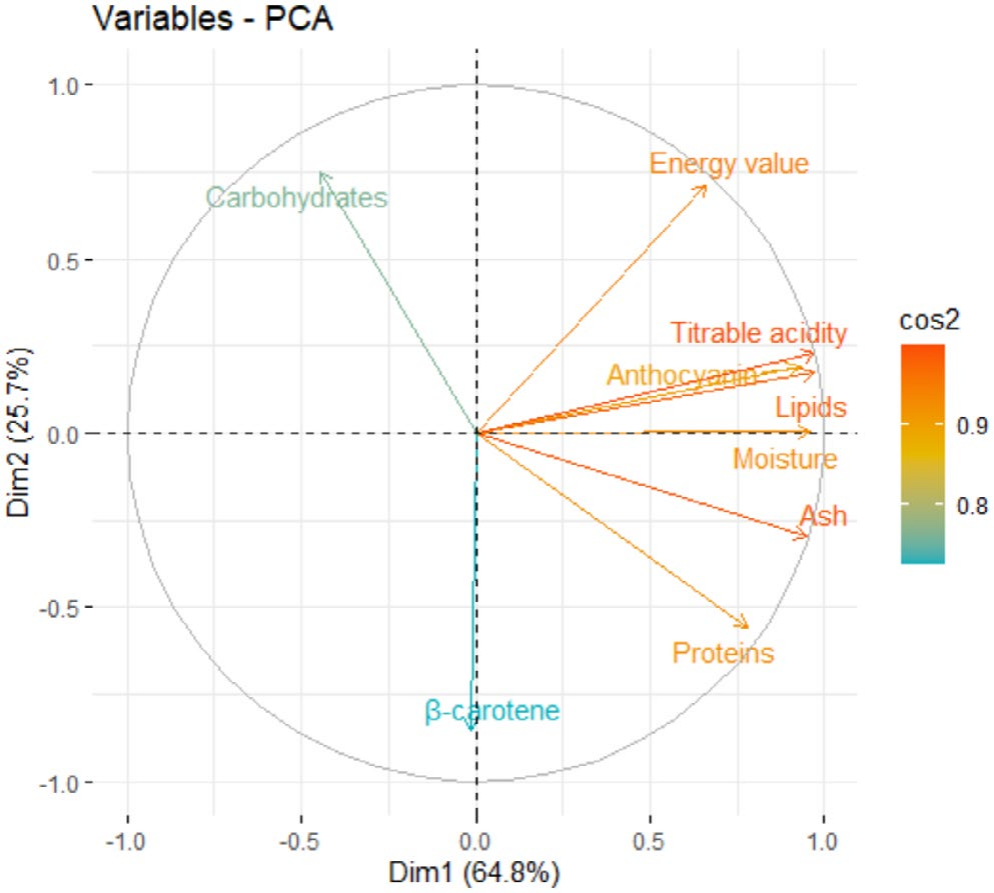
Principal component analysis of nutritional parameters.

**FIGURE 5:**
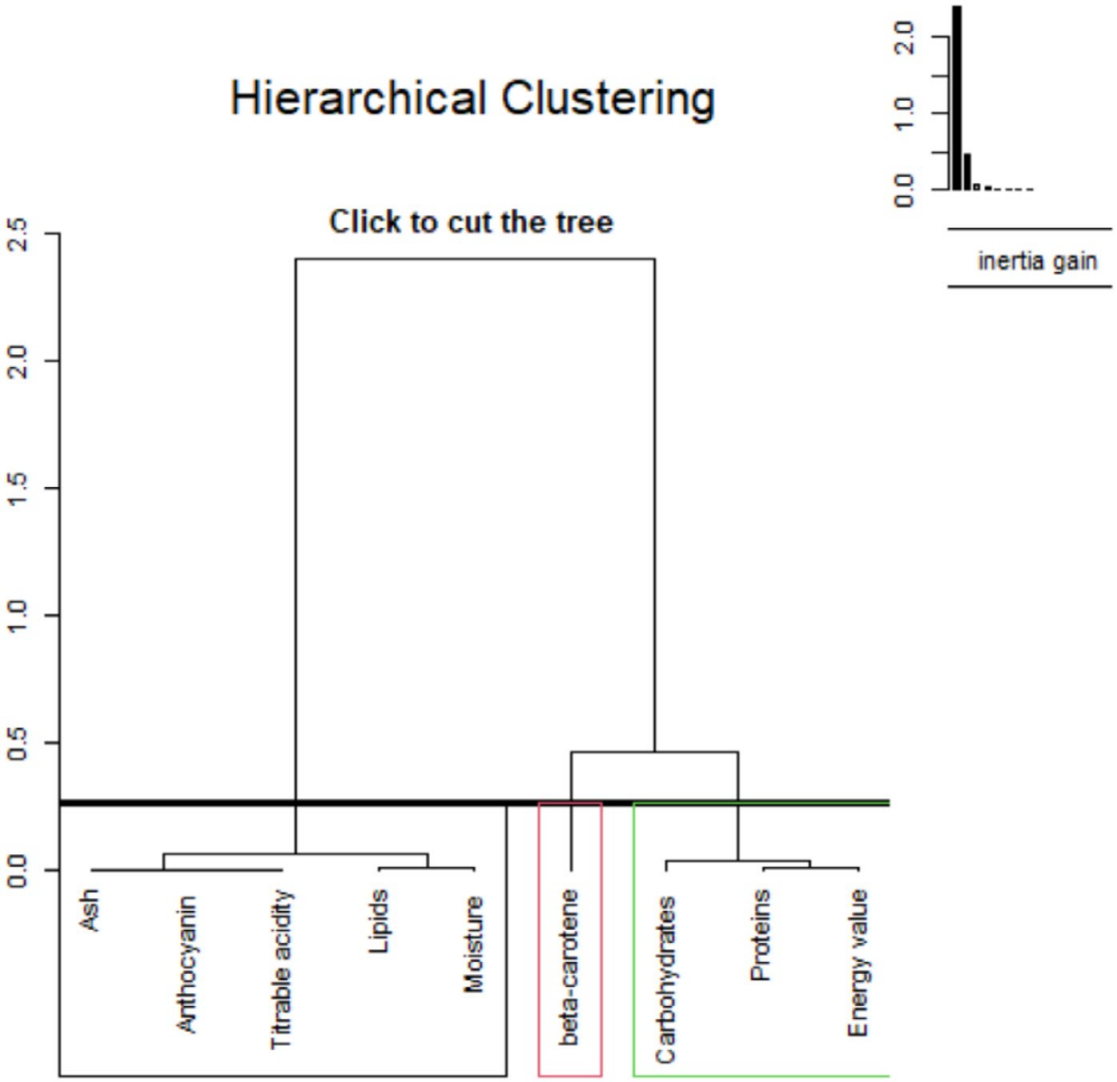
Dendrogram of the hierarchical classification of samples according to their physical and chemical characteristics.

Figures 4 and 5 show the projection of nutritional variables on the first two principal axes (Dim1 and Dim2) derived from PCA, which explain 64.8% and 25.7% of the total variance, respectively. The variables strongly correlated with the Dim1 axis are energy, titratable acidity, anthocyanins, lipids, moisture, ash, and protein, which are oriented in the same direction, suggesting a positive covariation between them. In contrast, β‐carotene shows a marked negative contribution to Dim1. The Dim2 axis is mainly influenced by carbohydrates, which are projected in the upper left part of the circle, indicating relative independence from the other variables. The quality of representation of the variables on the factorial plane is generally high (cos^2^ close to 1), particularly for lipids, energy, and anthocyanins, confirming their significant weight in the structuring of the data.

#### 3.2.1 Amino Acid Composition

**FIGURE 6:**
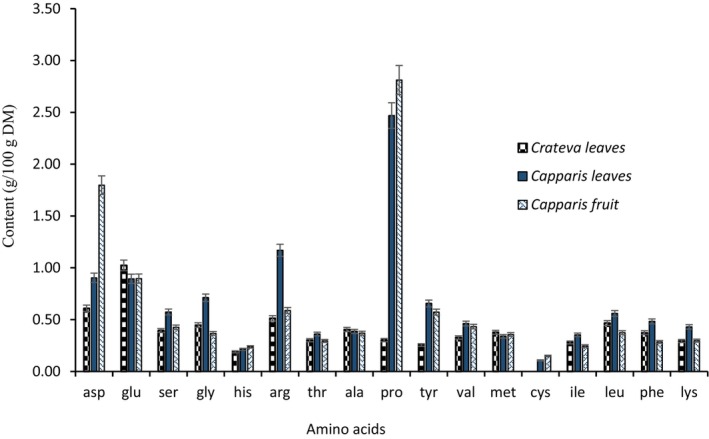
Amino acid profile of the different samples studied.

**FIGURE 7:**
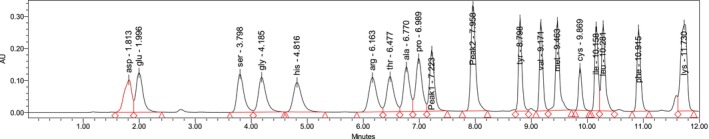
Chromatograms of amino acid profiles of *Crateva* leaves and *Capparis* fruits and leaves.

Figure 6 illustrates the free amino acid content (g/100 g DM) in *Crateva* leaves, *Capparis* leaves, and *Capparis* fruits. The most abundant amino acid is proline, with the highest content observed in *Capparis* fruit (3.02 g/100 g DM), followed by *Capparis* leaves (2.48 g/100 g DM) and *Crateva* leaves (2.31 g/100 g DM). Arginine is also highly represented in *Capparis* leaves (1.26 g/100 g DM), while it is much less abundant in the other samples. Aspartate is the second most abundant amino acid in *Capparis* fruit (1.82 g/100 g DM), compared to 1.05 g/100 g DM in *Capparis* leaves and 0.72 g/100 g DM in *Crateva* leaves. Amino acids such as glutamine, serine, glycine, and alanine are present in moderate concentrations, varying depending on the matrix. On the other hand, certain sulfur‐containing and aromatic amino acids, such as methionine, cysteine, phenylalanine, and tyrosine, are present in lower proportions (Figure 7). These data reveal a differentiated amino acid profile, with a notable richness in proline and amino acids of nutritional interest in *Capparis* fruits.

##### 3.2.1.1 Mineral Profile

**FIGURE 8:**
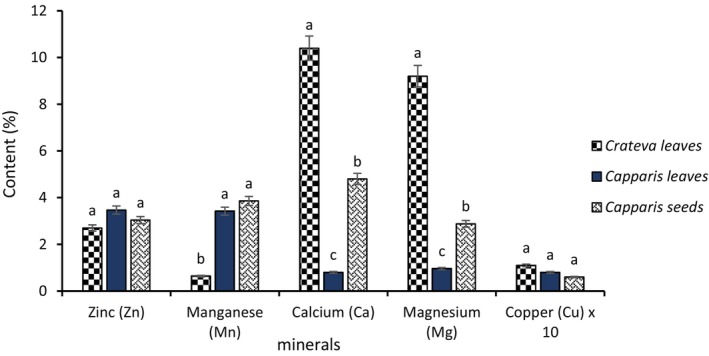
Mineral profile of *Crateva* leaves and *Capparis* fruits and leaves. The letters “a, b, c” indicate the different variations for each parameter. Sticks with the same letters do not have statistically different values. However, sticks with different letters have statistically different values.

The mineral profile of the samples analyzed (Figure 8) showed significant variations between *Crateva* leaves, *Capparis* leaves, and *Capparis* seeds. *Crateva* leaves had the highest calcium (10.4%) and magnesium (9.2%) contents, indicating a notable richness in essential macroelements. In contrast, *Capparis* seeds stood out for their high manganese concentration (3.86%), surpassing that observed in the other samples. Zinc is relatively well represented in all samples, with a slight predominance in *Crateva* leaves. Copper, although multiplied by a factor of 10 for better graphical visibility, remains weakly concentrated in all samples.

#### 3.2.2 Phenolic Compounds and Antioxidant Activity

The results reveal a significant variation (Pr > *F* < 0.001) in phenolic compound content and antioxidant activity among the different extracts and fractions tested (Table 1). The highest levels of flavonoids and total polyphenols are observed in fractions But2 (633.66 ± 15.79 mg EQ/100 g; 1636.88 ± 697.58 mg EAG/100 g) and especially But1 (403.00 ± 33.58 mg EQ/100 g; 3480.17 ± 509.04 mg EAG/100 g) fractions, highlighting the strong affinity of these compounds for n‐butanol. In terms of antioxidant activity, the Mac3 fraction has the best DPPH radical scavenging capacity (20.58 ± 0.72 μg/mL), while But1 and DCM2 also show notable activity (47.95 ± 4.26 and 49.27 ± 2.39 μg/mL, respectively). The highest FRAP values were recorded for DCM2 (118.98 ± 25.66 μg/mL), followed by But3 (102.20 ± 5.75 μg/mL), indicating good reducing power of these fractions. ABTS activity remained relatively stable for most fractions (~649–669 μg/mL), with the exception of Decoc1 and Decoc2, which had higher values (724.36 and 682.53 μg/mL), reflecting lower antioxidant capacity. These results highlight that fractions rich in polyphenols, particularly those obtained by butanol extraction, are the most effective in terms of antioxidant activity, regardless of the method used.

**TABLE 1:** Phenolic compound content and antioxidant activity. In each column, values sharing the same letter are not significantly different according to Tukey's HSD test at the 5% level.

Extracts and fractions	Flavonoids (mg EQ/100 g)	Polyphenols (mg EAG/100 g)	ABTS (μg/mL)	DPPH (μg/mL)	FRAP (μg/mL)
AC1	6.90 ± 0.51^ab^	79.95 ± 6.06^a^	650.39 ± 1.97^a^	80.59 ± 0.52^e^	91.70 ± 19.01^abc^
DCM1	87.67 ± 9.68^c^	248.19 ± 56.35^a^	669.20 ± 1.67^c^	58.52 ± 0.51^c^	80.17 ± 16.4^ab^
But1	403.00 ± 33.58^d^	3480.17 ± 509.04^g^	649.11 ± 1.14^a^	47.95 ± 4.26^b^	99.19 ± 6.19^bc^
Mac1	9.69 ± 2.52^ab^	290.98 ± 27.97^ab^	650.02 ± 1.14^a^	77.40 ± 1.57^de^	95.19 ± 7.23^bc^
Decoc1	2.35 ± 1.13^a^	72.26 ± 4.46^a^	724.36 ± 1.09^e^	80.76 ± 4.15^e^	85.10 ± 0.23^abc^
AC2	554.16 ± 59.44^e^	876.757 ± 41.53^cd^	649.84 ± 0.54^a^	75.39 ± 0.98^de^	99.51 ± 0.91^bc^
DCM2	12.89 ± 1.66^ab^	209.699 ± 12.60^a^	658.42 ± 8.13^ab^	49.27 ± 2.39^bc^	118.98 ± 25.66^c^
But2	633.66 ± 15.79^f^	1636.882 ± 697.58^e^	648.93 ± 2.21^a^	69.50 ± 7.91^d^	79.80 ± 3.25^ab^
Mac2	9.69 ± 27.97^ab^	1288.121 ± 187.64^e^	648.93 ± 0.83^a^	78.24 ± 0.63^de^	54.84 ± 4.48^a^
Decoc2	41.70 ± 2.56^b^	234.368 ± 27.38^a^	682.53 ± 5.40^d^	79.83 ± 0.57^e^	80.94 ± 10.30^ab^
AC3	22.69 ± 1.78^ab^	282.530 ± 5.47^a^	649.11 ± 1.14^a^	79.90 ± 1.06^e^	83.21 ± 7.24^abc^
DCM3	2.25 ± 0.20^a^	682.964 ± 26.18^bc^	667.37 ± 1.97^bc^	79.93 ± 0.10^e^	96.79 ± 9.46^bc^
But3	15.43 ± 3.56^ab^	2643.211 ± 54.46^f^	648.93 ± 0.31^a^	78.79 ± 0.74^de^	102.20 ± 5.75^bc^
Mac3	33.41 ± 1.51^ab^	1246.871 ± 95.38^de^	648.93 ± 0.83^a^	20.58 ± 0.72^a^	85.44 ± 9.78^abc^
Decoc3	7.17 ± 1.70^ab^	308.563 ± 19.21^ab^	674.32 ± 7.43^cd^	80.22 ± 7.62^e^	76.33 ± 10.14^ab^
Pr > F	0.000	0.000	0.000	0.000	0.000
Significatif	Oui	Oui	Oui	Oui	Oui

*Note:* 1: *Capparis corymbosa* leaves; 2: *Crateva adansonii* leaves; 3: *Capparis corymbosa* fruits. In each column values with the same letter (from a to f) are not significantly different according to Tukey’s test (HSD) at the 5% level.

Abbreviations: AC, ethyl acetate fraction; But, butanol fraction; DCM, dichloromethane fraction; Decoc, decocted extract; Mac, macerated extract.

#### 3.2.3 Anti‐Nutritional Factor and Anthocyanin Content

**FIGURE 9:**
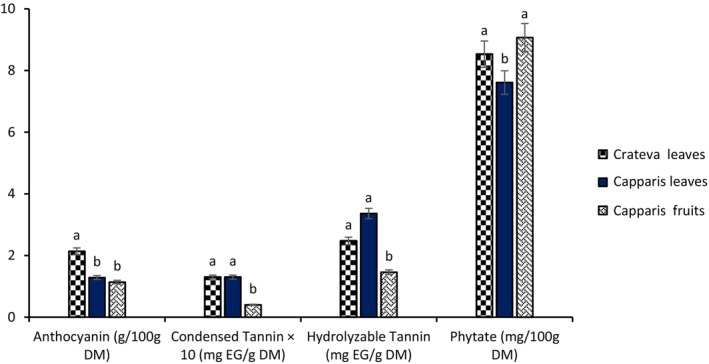
Anti‐nutritional factor content of *Crateva* leaves and *Capparis* fruits and leaves. The letters “a, b” indicate the different variations for each parameter. Sticks with the same letters do not have statistically different values. However, sticks with different letters have statistically different values.

Figure 9 shows the anti‐nutritional factor content of *Crateva* leaves, *Capparis* fruits, and *Capparis* leaves. *Crateva* leaves had the highest anthocyanin content (2.1 g/100 g DM), followed by *Capparis* fruits (1.29 g/100 g DM) and *Capparis* leaves (1.14 g/100 g DM). As for anti‐nutritional factors, condensed tannins showed similar values in *Crateva* leaves (1.3 mg EG/g DM) and *Capparis* leaves (1.3 mg EG/g DM), while the fruits contained lower amounts (0.4 mg EG/g DM). As for hydrolyzable tannins, a higher content was observed in *Capparis* leaves (3.36 mg EG/g DM), followed by *Crateva* leaves (2.47 mg EG/g DM) and *Crateva* fruits (1.46 mg EG/g DM). Phytates were found in large quantities in all samples, with a predominance in *Capparis* fruits (9.0 mg/100 g DM), followed by *Crateva* leaves (8.7 mg/100 g DM) and *Capparis* leaves (7.9 mg/100 g DM).

## 4 Discussion

The leaves of *Crateva adansonii* have a relatively high water, ash, titratable acidity, protein, and lipid content, highlighting their nutritional potential. In contrast, the fruits of *Capparis corymbosa* are mainly rich in carbohydrates, while its leaves have more moderate levels of these components. The relatively low water activity observed in *Crateva* leaves compared to previously reported values is particularly advantageous, as it improves the stability and shelf life of the product by limiting microbial growth and enzymatic reactions during storage (Ali et al. 2025; He et al. 2024). The ash content of *Crateva* leaves confirms a significant presence of mineral elements. This parameter, often used as an indirect indicator of mineral richness, can vary depending on several factors such as moisture content, soil characteristics, plant species, and varietal differences (Kumar et al. 2018; Buturi et al. 2021). These minerals play an essential role in maintaining the physiological and biochemical processes of the human body (Ali 2023). Titratable acidity, which is also high in *Crateva* leaves, contributes to the preservation and sensory quality of food products. It influences not only taste but also microbial stability, making it an essential factor in food quality control (Sharma et al. 2020). From a macronutrient perspective, *Crateva* leaves appear to be particularly rich in protein and lipids compared to previous reports. This composition highlights their nutritional importance, particularly given the role of protein in hormone and enzyme synthesis, as well as in supporting immune function and overall metabolic health (Alwarawrah et al. 2018). On the other hand, the high carbohydrate content of *Capparis* fruits suggests their potential as a valuable source of energy. Given their likely low glycemic index, they could be a healthier alternative to conventional carbohydrate sources, particularly for people who want to regulate their blood sugar levels (Sacks et al. 2014). Collectively, these results highlight the complementary nutritional profiles of *Crateva adansonii* and *Capparis corymbosa* , confirming their value as part of a diverse and balanced diet. Mineral analysis revealed particularly high levels of calcium and magnesium in the leaves of *Crateva adansonii*, compared to the leaves and fruits of *Capparis corymbosa* . In contrast, *Capparis* extracts are notable for their high concentrations of trace elements such as zinc and manganese, which are present in both leaves and fruits. These results highlight the mineral richness of both plant species, with a predominance of calcium and magnesium in *Crateva*, making it an interesting food resource for supporting bone health, enzyme regulation, and other physiological functions (Shaikh et al. 2017; Oli et al. 2024). Calcium, the body's main mineral, plays a role in cellular metabolism, blood clotting, enzyme activation, and bone and tooth formation (Ciosek et al. 2021). Magnesium, meanwhile, is essential for growth, proper immune system function, blood pressure regulation, and insulin secretion (Gill 2021). Its role is also recognized in the prevention of disorders such as cardiomyopathy and muscle degeneration (Al Alawi et al. 2018; Kirkland et al. 2018). Manganese, present in significant quantities in *Capparis corymbosa* , is an essential trace element for bone and amino acid synthesis. It also has anti‐inflammatory and antioxidant properties, helping to protect the body against free radicals. A manganese deficiency can lead to fertility problems, glucose metabolism disorders, and immune system alterations (Li and Yang 2018). According to (Annaz et al. 2022) the *Capparis* genus is generally rich in essential minerals such as calcium, iron, potassium, phosphorus, magnesium, zinc, and manganese, all of which are involved in maintaining optimal metabolic functions. In addition, *Capparis* capers are traditionally recognized for their high vitamin and fiber content and low fat and calorie content (Nirmala et al. 2022). The amino acid concentrations detected in the leaves and fruits of *Capparis corymbosa* , as well as in the leaves of *Crateva adansonii*, reveal a rich and diverse profile. Nineteen amino acids were identified in the leaves and fruits of *Capparis*, compared to eighteen in the leaves of *Crateva*, including nine essential amino acids such as lysine, histidine, threonine, valine, methionine, phenylalanine, leucine, and isoleucine. These amino acids are essential for the proper functioning of the body, particularly in the synthesis of enzymes and hormones and in maintaining cell structures (Holeček 2022). As major constituents of body mass after water, their intake through food is crucial (Tessari et al. 2016). Thus, the high amino acid content, particularly essential amino acids, gives these two species high nutritional potential and justifies their use as high‐value‐added food sources. From a nutritional standpoint, the high mineral and amino acid content of *Crateva adansonii* and *Capparis corymbosa* highlights their potential role in meeting daily dietary requirements. For example, the high calcium and magnesium content of *Crateva adansonii* leaves could contribute significantly to bone health, enzyme regulation, and cardiovascular function. Similarly, the high concentrations of trace elements such as zinc and manganese in *Capparis corymbosa* play an essential role in immune defense, antioxidant protection, and reproductive health (Nirmala et al. 2022). The abundance of essential amino acids in both species further enhances their nutritional value, providing elements that are indispensable for protein synthesis and metabolic regulation (Holeček 2022). In practical terms, incorporating moderate amounts of these plants into the diet could help meet daily nutritional needs. For example, 100 g of dried *Crateva adansonii* leaves could provide a significant portion of the recommended daily allowance (RDA) of calcium and magnesium, while the carbohydrate‐rich fruits of *Capparis corymbosa* could serve as a natural energy source with potential benefits for glycemic control (Rathinavel et al. 2021). Thus, the complementary nutritional profiles of these two species not only reinforce their value as food resources but also justify their inclusion in preventive nutritional strategies aimed at combating micronutrient deficiencies and promoting metabolic health (Ali 2023). Extracts and fractions derived from the leaves of *Crateva adansonii* and the leaves and fruits of *Capparis corymbosa* were found to be particularly rich in total phenolic compounds and flavonoids. Butanolic fractions, in particular, had the highest concentrations, suggesting strong potential antioxidant activity. Compared to data reported for other species of the genus *Capparis*, such as *Capparis spinosa* (Wojdyło et al. 2019; Mollica et al. 2019), our results show higher levels of these bioactive compounds, probably due to differences between the species studied or the analytical standards used for the assay. The abundance of secondary metabolites in these two plant species is a major asset for their potential use in the prevention or mitigation of chronic diseases. Flavonoids, which are abundant in various parts of the plants, are known for their powerful antioxidant properties (Wojdyło et al. 2019; Górniak et al. 2019). They play a protective role against oxidative stress by neutralizing free radicals, thereby helping to prevent degenerative diseases such as diabetes, cancer, inflammation, and cardiovascular disorders. The *Capparis* genus is well documented for its antioxidant, antifungal, anti‐inflammatory, hepatoprotective, and antidiabetic effects (Annaz et al. 2022). As for *Crateva adansonii*, several studies highlight its medicinal and nutritional potential, particularly its antioxidant and antibacterial activities linked to the diversity of its secondary metabolites (Rathinavel et al. 2021; Nounagnon et al. 2018). Tannins, also detected in the analyzed extracts, are known for their therapeutic role. Present in condensed or hydrolyzable form, they have a wide range of biological activities, including antibacterial, antioxidant, anti‐hemorrhagic, and antiviral effects (Sahakyan et al. 2020). However, their affinity for proteins can reduce digestibility, which requires particular attention to their concentration in the diet. Despite this, moderate consumption is associated with a reduction in blood pressure and an improvement in the lipid profile (Kaspchak et al. 2019). In addition, the analyses revealed significant concentrations of phytates, with a predominance observed in *Capparis corymbosa* fruits. Although considered anti‐nutritional factors due to their ability to complex certain minerals, phytates also have beneficial health effects. They are recognized for their antioxidant and antidiabetic properties and their potential role in preventing the formation of renal stones (Pujol et al. 2023; Pires et al. 2023). Thus, the bioactive composition of *Crateva adansonii* and *Capparis corymbosa* extracts reinforces their value not only as functional food sources but also as promising candidates in the fields of phytotherapy and preventive nutrition.

## 5 Conclusion

This study revealed the high nutritional and bioactive potential of *Crateva adansonii* and *Capparis corymbosa* . The leaves of *C. adansonii* have high levels of protein, minerals, and essential amino acids, while the fruits of *C. corymbosa* are rich in carbohydrates, minerals, and phytates. The extracts, particularly the butanolic fractions, are rich in phenolic and flavonoid compounds, with notable antioxidant activity (DPPH, ABTS, FRAP). These results position these species as promising sources of functional compounds of nutraceutical and phytotherapeutic interest. Further research, including in vivo and clinical studies, as well as the evaluation of sustainable valorization processes, will confirm their effectiveness and promote their integration into food and medical applications.

## Author Contributions

**Kabakdé Kaboré:** formal analysis, writing – review and editing. **Frédéric Anderson Konkobo:** methodology, writing – review and editing, validation. **Mamoudou Hama Dicko:** visualization, writing – review and editing, supervision. **Abdoudramane Sanou:** formal analysis, writing – review and editing. **Edwige Noëlle Roamba:** methodology, writing – review and editing, validation. **Poussian Raymond Barry:** formal analysis, writing – review and editing. **Adama Pamba Séré:** formal analysis, writing – review and editing. **Roger Dakuyo:** formal analysis, writing – review and editing. **Kiessoun Konaté:** visualization, writing – review and editing, supervision.

## Conflicts of Interest

The authors declare no conflicts of interest.

## Data Availability

The data that support the findings of this study are available from the corresponding author upon reasonable request.
